# A Comparative Study Between ECG- and PPG-Based Heart Rate Sensors for Heart Rate Variability Measurements: Influence of Body Position, Duration, Sex, and Age

**DOI:** 10.3390/s25185745

**Published:** 2025-09-15

**Authors:** Alexandre Coste, Geoffrey Millour, Christophe Hausswirth

**Affiliations:** 1 BeScored Institute, 06560 Valbonne, France; 2 Laboratoire Motricité, Interactions, Performance, MIP, UR 4334, Nantes Université, 44109 Nantes, France; 3 Inserm CAPS UMR 1093, UFR STAPS, Université Bourgogne Europe, 21078 Dijon, France

**Keywords:** autonomic nervous system, RMSSD, SDNN, photoplethysmography, electrocardiography, wearable sensors

## Abstract

This study evaluated the validity of a photoplethysmography (PPG)-based sensor (Polar OH1) for measuring heart rate variability (HRV), compared to an electrocardiography (ECG)-based reference device (Polar H10), considering body position (supine vs. seated), recording duration (2 vs. 5 min), sex, and age (≤40 vs. >40 years). HRV parameters (RMSSD and SDNN) were analyzed in 31 healthy adults using intraclass correlation coefficients (ICCs) and Bland–Altman analyses. Excellent reliability was observed between the devices in the supine position (RMSSD: ICC = 0.955; SDNN: ICC = 0.980), and good to excellent reliability in the seated position (RMSSD: ICC = 0.834; SDNN: ICC = 0.921). Mean biases ranged from −2.1 ms to −8.1 ms, with wider limits of agreement in the seated condition. The change in posture from supine to seated resulted in moderate reliability for both metrics, regardless of the device. Only marginal differences were found between 2- and 5-min recordings. Moreover, agreement was less consistent in older participants and females, suggesting potential effects of age and sex on signal quality. These findings support the use of PPG-based devices for short-term HRV assessment at rest, while highlighting the importance of considering posture, age, and sex when interpreting the results.

## 1. Introduction

In recent years, photoplethysmography (PPG) sensors gained widespread popularity for monitoring heart rate (HR) and heart rate variability (HRV) in wearable devices such as smartwatches and fitness trackers [1]. Unlike electrocardiography (ECG)-based chest straps, which directly measure the heart’s electrical activity, PPG relies on optical sensors to estimate HRV by detecting blood volume changes in the peripheral circulation, typically from the wrist or forearm [2]. This approach makes HRV monitoring more accessible and convenient for applications such as lifestyle management, stress assessment, and athletic performance monitoring [1,3,4,5]. However, despite these advantages, comparative studies evaluating the accuracy and reliability of PPG-based HRV measurements remain limited, particularly when compared to ECG-based measurements, which are considered the gold standard [6].

A fundamental difference between ECG and PPG lies in their measurement mechanisms and recording sites. ECG-based sensors capture the heart’s electrical activity directly from the chest, providing precise R–R intervals that enable accurate HRV analysis. In contrast, PPG sensors estimate HRV by detecting peripheral blood volume fluctuations, which are influenced by several factors, such as vascular compliance, pulse arrival time (PAT), pulse transit time (PTT), and microcirculatory regulation [7,8]. As a result, PPG-derived HRV—often referred to as pulse rate variability (PRV)—may differ from ECG-derived HRV due to variations in pulse wave propagation and autonomic regulation at peripheral sites. To such an extent, some authors argue that PRV should be considered a distinct biomarker rather than a surrogate for HRV [9,10].

Several factors may influence the accuracy and comparability of HRV measurements obtained through ECG and PPG. One critical factor is body position. Previous research has shown that autonomic nervous system (ANS) activity varies across postural conditions, with the supine position favoring parasympathetic dominance, while seated or upright positions are associated with increased sympathetic activity [2,6]. PPG has been reported to overestimate parasympathetic activity, particularly in non-supine positions, due to variability in pulse arrival time (PAT) and pulse transit time (PTT) [11,12]. As a result, differences in body position may amplify discrepancies between HRV metrics derived from PPG and those obtained from ECG.

Another important factor influencing HRV measurements is the duration of the recording. Standard HRV assessments typically rely on 5 min recordings, as recommended by the Task Force of the European Society of Cardiology and the North American Society of Pacing and Electrophysiology [13]. However, ultra-short-term recordings (e.g., 2 min) have been proposed as a more practical alternative for use in applied settings [14]. While frequency domain and non-linear HRV metrics can often be reliably estimated from shorter recordings, time domain parameters such as the root mean square of successive differences (RMSSD), and the standard deviation of normal-to-normal intervals (SDNN) generally require longer durations to ensure accuracy [11,13]. Moreover, shorter recordings are more vulnerable to noise and motion artifacts, especially when using PPG-based sensors, as peripheral blood flow is highly sensitive to external disturbances [15,16]. It is therefore recommended to perform HRV measurements at rest.

Individual factors, such as age and sex, also play a key role in HRV variability and measurement accuracy. HRV tends to decline with age due to reduced autonomic flexibility, with time domain metrics such as RMSSD and SDNN showing a gradual decrease [17,18]. For example, [18] analyzed 24 h ECG recordings from 1743 subjects aged 40 to 100 years and observed a linear decline in SDNN. Interestingly, RMSSD followed a U-shaped pattern, decreasing between ages 40 and 60 before increasing again after 70, suggesting complex interactions between aging and autonomic function. Similarly, [19] found that the most significant HRV reductions occur between the second and third decades of life. Sex-related differences in HRV have also been extensively documented. Females generally exhibit greater parasympathetic activity, which influences HRV parameters and results in shorter R–R intervals compared to males [20]. This relative vagal dominance in females has been linked to cardiovascular protective effects but may also lead to differences in HRV measurement reliability across sensor modalities. In contrast, males tend to show greater sympathetic dominance, which can cause more pronounced discrepancies in HRV parameters between ECG and PPG due to their differing sensitivities to autonomic fluctuations [2,19]. Additionally, vascular properties, such as arterial stiffness and endothelial function, which vary between males and females, may further affect PPG-derived HRV measurements by influencing pulse wave propagation and peripheral circulation dynamics [21].

In light of these considerations, the aim of this study is to investigate differences in HRV parameters, specifically RMSSD and SDNN, obtained from ECG and PPG signals. We assess how body position (supine vs. seated), measurement duration (2 min vs. 5 min), and individual factors, such as age and sex, affect the comparability of HRV measures between these two modalities. By accounting for these variables, we aim to improve our understanding of the reliability of PPG-based HRV and its applicability in different populations and settings.

## 2. Materials and Methods

### 2.1. Participants

Thirty-one healthy participants were recruited for the study. Their characteristics, including age, height, and body mass, are summarized in Table 1. Inclusion criteria required participants to be between 18 and 70 years old, free of known heart conditions or diseases, and without hypertension. Additionally, due to the influence of skin pigmentation on PPG measurements [22], only individuals with Fitzpatrick skin phototypes I, II, or III were included. Exclusion criteria were regular use of medications affecting the cardiovascular or endocrine systems, current smoking, or pregnancy. All participants received detailed information about the study’s purpose, procedures, potential risks, and benefits, and provided written informed consent prior to participation. The study protocol was approved by the National Ethics Committee (ethical approval: IRB00012476-2024-13-11-352) and conducted in accordance with the 2024 Declaration of Helsinki [23].

### 2.2. Apparatus

Two commercially available devices were used to collect HRV data during the study:

Polar H10 Chest Strap

The Polar H10 (Polar Electro Oy, Kempele, Finland) is a high-precision chest strap heart rate monitor widely used in sports and research for HRV analysis [24]. Equipped with two electrodes embedded in the chest strap, it detects the electrical signals generated by the heart with each beat. These signals are used to calculate the R–R intervals, which represent the time intervals between successive R-wave peaks in the ECG signal. R–R intervals are essential for HRV analysis, as they reflect the autonomic nervous system’s regulation of cardiac function. In this study, raw R–R interval data were recorded using the Elite HRV app (Elite HRV, Inc., Asheville, NC, USA) and analyzed with the MATLAB software (R2022a, The MathWorks, Natick, MA, USA).

Polar OH1 PPG Sensor

The Polar OH1 (Polar Electro Oy, Kempele, Finland) is a wearable heart rate sensor that uses PPG technology to measure HR by detecting changes in blood volume. It has been validated in sports settings, demonstrating high accuracy for heart rate measurement, particularly when chest and arm movements are limited [25]. Worn on the upper arm, it employs the same PPG technology found in many modern fitness trackers and smartwatches, utilizing green LEDs. In this study, the peak-to-peak interval (PPI) mode of the Polar OH1 was activated using the official Polar Software Development Kit (SDK, https://github.com/polarofficial/polar-ble-sdk accessed on 4 November 2024), enabling the extraction of pulse rate variability (PRV) data. The sensor was connected via Bluetooth to a custom-built web application developed in JavaScript using the Web Bluetooth API, which enabled real-time acquisition and storage of PPG data. To minimize interference from arm movements, the Polar OH1 was worn on the non-dominant arm (i.e., left forearm for right-handed participants and right forearm for left-handed participants). This placement was chosen to avoid any potential movement-related interference, even though all measurements were taken at rest in a static position.

### 2.3. Study Design and Procedures

We employed a cross-over design where all participants completed both seated and supine conditions in a randomized order. Data were collected from December 2024 to March 2025, between 11:00 a.m. and 7:00 p.m. Testing was conducted on a massage table for the supine position and a comfortable office chair for the seated position. All measurements took place in a controlled environment (quiet, dark room) to minimize sensory interference. Participants were instructed to relax, breathe normally, and keep their eyes closed throughout the 5 min measurement sessions, each preceded by a 1 min stabilization phase to allow heart rate to return to baseline. The Polar H10 and Polar OH1 sensors were synchronized for simultaneous recording, enabling direct comparison of ECG- and PPG-based HRV measurements.

### 2.4. Data Analysis

All data were stored in an electronic database, then preprocessed and analyzed using MATLAB and Microsoft Excel (Redmond, Washington, DC, USA). Time domain HRV metrics, including RMSSD and SDNN, were extracted from both the Polar H10 and Polar OH1 devices using the HRVTool MATLAB toolbox [26,27]. Two filters were tested [26,28] to identify the most accurate method for processing R–R intervals. We selected the HRV.RRfilter function, which minimizes fluctuations exceeding 15% of the previous interval, helping to remove artifacts while preserving physiologically relevant HRV variations [26]. To compare HRV parameters between devices and conditions, we used intraclass correlation coefficients (ICCs), mean absolute error (MAE), root mean square error (RMSE), and Bland–Altman analysis. ICCs were interpreted according to [29]: <0.50 = poor, 0.50–0.75 = moderate, 0.75–0.90 = good, and >0.90 = excellent reliability. Bland–Altman analysis calculated mean differences and 95% limits of agreement (LoA). Measurements were analyzed over 2 and 5 min intervals, with primary focus on the 5 min window to enable comparisons by sex (male vs. female) and age group (≤40 years vs. >40 years). The 5 min duration is standard for short-term HRV assessment, providing stable results during normal breathing [6]. The 2 min segment was taken from the middle of the 5 min recording, starting 1 min 30 s after onset and ending 1 min 30 s before completion, to avoid artifacts typically present at the beginning (due to stabilization) and the end (due to anticipatory movements) of measurements [30].

## 3. Results

### 3.1. Participant Characteristics

Table 1 shows the general characteristics of our study sample. Descriptive statistics are provided for age, height, and body mass.

**Table 1:** General participant characteristics and subgroup breakdown by age (≤40 vs. >40) and sex (male vs. female). Values are presented as mean ± standard deviation, with minimum and maximum values in brackets. n: number of participants.

	Overall Participants (n = 31)	
**Sex (number)**	Female: 18—Male: 13	
**Age (years)**	43 ± 12 [21–66]	
**Height (m)**	1.71 ± 0.09 [1.50–1.89]	
**Body mass (kg)**	72 ± 16 [46–115]	
	**≤40 years old (n = 14)**	**>40 years old (n = 17)**
**Sex (number)**	Female: 8—Male: 6	Female: 10—Male: 7
**Age (years)**	33 ± 5 [21–40]	52 ± 7 [41–66]
**Height (m)**	1.73 ± 0.10 [1.59–1.89]	1.69 ± 0.09 [1.50–1.85]
**Body mass (kg)**	77 ± 19 [50–115]	67 ± 13 [46–89]
	**Females (n = 18)**	**Males (n = 13)**
**Age (years)**	44 ± 12 [23–66]	43 ± 12 [21–63]
**Height (m)**	1.65 ± 0.06 [1.50–1.75]	1.78 ± 0.07 [1.64–1.89]
**Body mass (kg)**	67 ± 18 [46–115]	78 ± 12 [62–100]

### 3.2. Impact of Sensor Type on HRV Parameters

**Figure 1:**
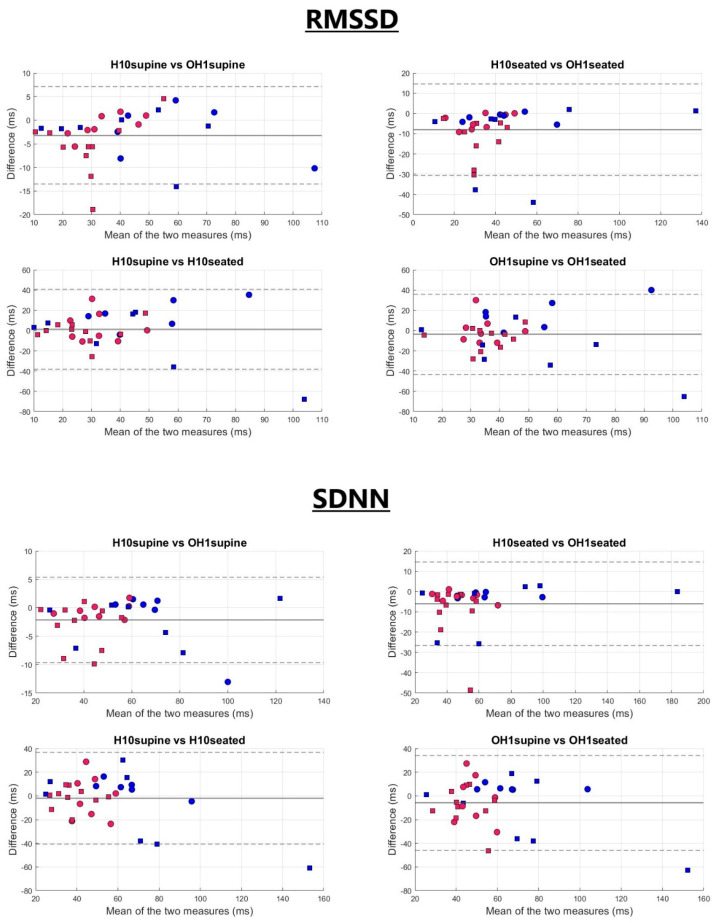
Bland–Altman analysis of RMSSD and SDNN across the different body position conditions (supine and seated) and devices (H10 vs. OH1). The continuous grey line represents the bias, while the dashed grey lines indicate the upper and lower limits of agreement. Markers indicate participant groups: circles for participants aged ≤40 years, squares for participants aged >40 years; blue for men and pink for women.

The comparison between the two sensors demonstrated good agreement for HRV measurements across both recording durations, particularly in the supine position (see Figure 1 and Table 2). For RMSSD, ICCs (3,1) between the Polar H10 and OH1 were good to excellent in the supine position (ICC = 0.955 for 5 min; 0.869 for 2 min) and remained good in the seated position (ICC = 0.834 for 5 min; 0.868 for 2 min). Mean differences between the H10 and OH1 were relatively small when supine (−3.21 ms for 5 min; −2.91 ms for 2 min), increasing slightly in the seated position (−8.05 ms for 5 min; −6.14 ms for 2 min). A similar pattern was observed for SDNN, with excellent ICCs (3,1) in both supine (ICC = 0.980 for 5 min; 0.929 for 2 min) and seated (ICC = 0.921 for 5 min; 0.916 for 2 min) positions. Mean differences for SDNN were small in the supine position (−2.11 ms for 5 min; −2.63 ms for 2 min) and slightly larger in the seated position (−6.02 ms for 5 min; −5.23 ms for 2 min), accompanied by wider limits of agreement (LoA). MAE and RMSE values mirrored these trends, with lower errors observed in the supine position compared to seated.

### 3.3. Impact of Body Position on HRV Parameters

When comparing the effect of body position, HRV values remained generally consistent between the supine and seated conditions for both sensors, although reliability decreased and variability increased. ICCs (3,1) for the OH1 were slightly lower than those for the H10 and fell within the moderate range (RMSSD 5 min: 0.560 vs. 0.608; SDNN 5 min: 0.674 vs. 0.728). A similar pattern was observed for 2 min recordings, with ICCs (3,1) dropping into the poor-to-moderate range (RMSSD 2 min: 0.468 vs. 0.482; SDNN 2 min: 0.507 vs. 0.621). Notably, SDNN consistently showed higher reliability than RMSSD across all durations and sensors. Mean differences between body positions were small to moderate for both sensors (−5.32 ms to 1.35 ms for H10; −7.92 ms to −1.89 ms for OH1), but the limits of agreement remained wide across devices, regardless of HRV metric or recording duration. MAE and RMSE values between postures followed a similar trend, with slightly larger errors observed for the OH1, particularly in the 2 min condition for SDNN. Nonetheless, the magnitude of error remained comparable between RMSSD and SDNN.

### 3.4. Influence of Age on HRV Parameters

**Figure 2:**
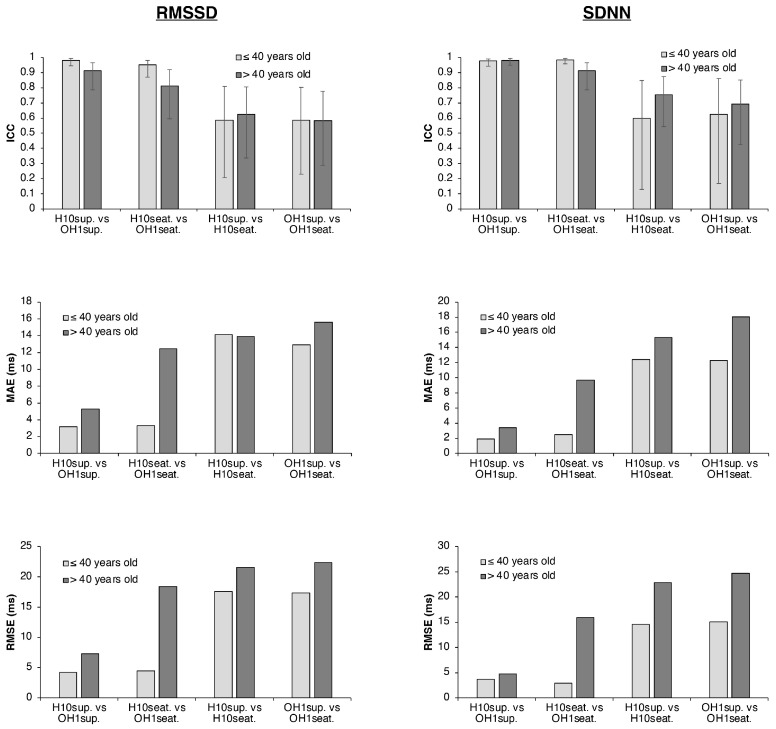
Bar plots of ICC, MAE, and RMSE for RMSSD and SDNN according to age groups (≤40 years vs. >40 years) across different body position conditions (supine and seated) and devices (H10 vs. OH1). Light gray bars represent participants aged ≤40 years and dark gray bars represent participants aged >40 years. Error bars on ICC indicate 95% confidence intervals.

When comparing HRV parameters between age groups (≤40 vs. >40 years), a general decline in RMSSD and SDNN values was observed with increasing age (see Figure 2 and Table 3). For RMSSD, ICCs (3,1) between the H10 and OH1 were good to excellent across both age groups and body positions (ranging from 0.812 to 0.981), though slightly lower in the seated position and among older participants. Mean differences between sensors were greater in the seated than in the supine position for both younger (−3.31 ms vs. −1.94 ms) and older participants (−12.08 ms vs. −4.47 ms), with wider limits of agreement (LoA) in the older group. MAE and RMSE values were also higher among older participants, particularly in the seated condition. For SDNN, ICCs (3,1) remained excellent across positions and age groups (0.912–0.984), yet mean differences and LoA were again larger in the older group. MAE and RMSE followed the same pattern, with higher errors observed in older participants and in the seated position. When comparing body positions, RMSSD values were relatively consistent between supine and seated conditions across age groups and sensors, with moderate to good reliability (ICCs: 0.581–0.623). However, mean differences, LoA, MAE, and RMSE were all higher in the older group. For SDNN, ICCs between positions were lower in the younger group than in the older group (0.598 and 0.624 vs. 0.754 and 0.693 for H10 and OH1, respectively), though younger participants displayed smaller mean differences, narrower LoA, and lower MAE and RMSE values.

### 3.5. Influence of Sex on HRV Parameters

**Figure 3:**
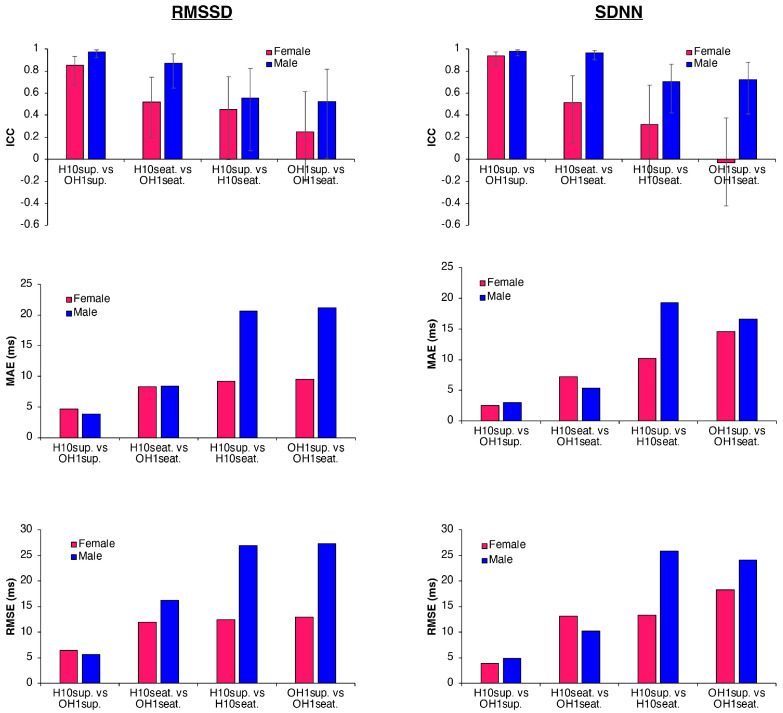
Bar plots of ICC, MAE, and RMSE for RMSSD and SDNN according to sex (male vs. female) across different body position conditions (supine and seated) and devices (H10 vs. OH1). Pink bars represent women and blue bars represent men. Error bars on ICC indicate 95% confidence intervals.

When comparing HRV parameters between sexes, females exhibited lower absolute RMSSD and SDNN values than males in both supine and seated positions (see Figure 3 and Table 4). For RMSSD, ICCs (3,1) between H10 and OH1 in the supine position were good for females and excellent for males (0.851 vs. 0.974), with mean differences of −3.76 ms and −2.46 ms, respectively. In the seated position, ICCs were lower, particularly among females (0.521 vs. 0.870), and mean differences increased in both groups (−8.27 ms for females vs. −7.74 ms for males). MAE and RMSE values were higher in the seated condition but remained similar between sexes. Comparable patterns were observed for SDNN, with excellent ICCs in the supine position for both females and males (0.939 vs. 0.980). In the seated position, reliability decreased in females (ICC = 0.513) but remained excellent in males (ICC = 0.967). As with RMSSD, MAE and RMSE values were higher in the seated condition, with similar magnitudes of error across sexes. Regarding the effect of body position, RMSSD showed reduced reliability in females, with poor ICCs between supine and seated positions for both sensors (0.450 for H10 and 0.251 for OH1). In contrast, ICCs in males remained moderate (0.555 for H10 and 0.524 for OH1). For SDNN, reliability between positions was higher in males (ICC = 0.702 for H10 and 0.720 for OH1) than in females (ICC = 0.317 for H10 and −0.031 for OH1). Despite relatively small mean differences across positions and sexes (ranging from −8.27 to 2.09 ms), LoA remained wide, occasionally exceeding ±50 ms. MAE and RMSE values confirmed this variability, with consistently larger errors in the seated condition.

## 4. Discussion

The aim of this study was to assess the validity of PPG-based HRV measurements in comparison with ECG-based measurements, and to examine the influence of recording duration, body position, age, and sex on measurement accuracy.

Our main findings indicate generally good to excellent agreement between the ECG-based Polar H10 and the PPG-based Polar OH1 for the time domain HRV parameters RMSSD and SDNN in healthy individuals. This agreement was consistent across both supine and seated positions, supporting previous research demonstrating the reliability of PPG technology for HRV assessment under controlled conditions [5]. Furthermore, recording duration had a limited impact on measurement accuracy. Although shorter recordings introduced slightly greater variability, our results are consistent with prior studies indicating that 2 min recordings can provide sufficiently accurate RMSSD and SDNN values [14]. This finding is particularly relevant for practical applications, where longer recordings may be impractical. However, ensuring that participants are in a stable physiological state before measurement remains essential to ensure data validity [13]. 

A more detailed analysis revealed that agreement between sensors varied depending on body position. Specifically, concordance between PPG- and ECG-derived measures was higher in the supine position, but significantly lower in the seated position. These discrepancies were reflected by larger errors, greater mean differences, and wider limits of agreement, particularly for PPG-derived measurements. This suggests that sensor agreement is reduced under certain physiological conditions associated with posture. Furthermore, within-sensor comparisons between supine and seated positions also showed consistently lower agreement, indicating that the observed discrepancies reflect genuine physiological changes rather than sensor inaccuracies alone. The transition from supine to seated posture is known to increase sympathetic activity and reduce parasympathetic tone [11] resulting in distinct autonomic states and, consequently, reduced agreement between positions. Several factors may account for this increased variability. First, PTT introduces fluctuations in PRV that are not directly related to cardiac autonomic modulation [31,32]. These fluctuations—driven by respiration-induced changes in intrathoracic pressure—may be amplified in the seated position due to postural effects on vascular dynamics. Second, the accuracy of beat-to-beat interval extraction from PPG signals is limited, particularly in individuals with increased vascular stiffness or altered pulse wave morphology. For example, reflected waves can distort peak detection in older adults, reducing the reliability of HRV estimates [33]. These limitations primarily affect short-term HRV indices, such as RMSSD and SDNN, although alternative approaches, such as valley-to-valley interval detection, have shown promise in improving accuracy [33]. Together, these findings highlight the importance of considering both sensor type and body position when interpreting HRV data.

When considering the effects of age, our analysis of HRV parameters over a 5 min recording period revealed a general decline in RMSSD and SDNN values with increasing age, which is consistent with previous findings [17,18]. This reduction in HRV among older participants reflects an age-related decrease in autonomic nervous system adaptability [19]. However, prior research suggests that the decline in RMSSD with age does not follow a strictly linear trajectory, but rather a U-shaped pattern, with a slight increase observed beyond the age of 70 [18]. Since our study included only participants aged 18 to 70 years, we were unable to explore this potential non-linear trend. Moreover, the agreement between HRV values derived from the OH1 and H10 was generally lower in participants over 40 years of age, regardless of body position. This may be partially explained by age-related increases in arterial stiffness. In older individuals, the reflected wave in the PPG signal becomes more pronounced, potentially complicating peak detection and reducing the accuracy of HRV estimation [33]. Sex-related differences were also observed, with females displaying lower absolute RMSSD and SDNN values compared to males. These results align with previous literature indicating that women tend to have higher resting heart rates and lower overall HRV, likely due to differences in autonomic regulation [20]. Additionally, the accuracy of PPG-derived HRV measures was slightly reduced in females, particularly in the seated position. This may be attributed to sex-related differences in vascular compliance and endothelial function, which affect pulse wave dynamics and thus the accuracy of PRV estimation [19,21]. Hormonal fluctuations may also contribute to increased intra-individual variability in HRV among females, potentially affecting agreement between ECG- and PPG-based measurements [34]. Nevertheless, given the relatively small sample size, these subgroup-specific observations should be interpreted with caution.

Despite its strengths, this study has several limitations that should be considered when interpreting the findings. First, our sample included only healthy participants aged 18 to 70 years, limiting the generalizability to younger, older, or clinical populations. Additionally, the participants had light skin tones (Fitzpatrick phototypes I–III), which may affect the applicability of results to individuals with darker skin. Indeed, recent research [22] suggests that green-light PPG sensors such as the Polar OH1 may show reduced accuracy in darker skin due to increased light absorption and scattering. Secondly, we focused solely on two time domain HRV metrics, RMSSD and SDNN, commonly used in consumer devices. Although informative, these parameters do not capture the full complexity of autonomic regulation. Future studies should include additional indices, especially frequency domain measures (e.g., LF, HF, and LF/HF ratio), for a more comprehensive evaluation of PPG accuracy. Another limitation is the lack of repeated measurements within each body position. HRV exhibits intra-individual variability even under stable conditions, making it difficult to fully isolate the effect of posture from natural fluctuations. While we randomized the order of recordings and included rest periods to mitigate this, repeated measures would strengthen estimates of measurement reliability and better attribute differences to posture. Future research should address these issues by including more diverse populations, assessing a broader range of HRV parameters, and incorporating repeated recordings. Moreover, applying machine learning techniques to long-term HRV data could reveal health-related trends over time, building upon the foundational validation of PPG-based HRV accuracy provided here.

## 5. Conclusions

The present findings support the use of PPG-based HRV monitoring for practical assessments in healthy individuals, demonstrating good agreement with ECG-based sensors across conditions. However, as the data were collected exclusively from healthy participants with Fitzpatrick skin phototypes I–III, the results may not be fully generalizable to the wider population. Furthermore, while the observed postural differences likely reflect physiological variations, these findings may not extend to all body positions or PPG sensor types.

## Figures and Tables

**Table 2:** Comparison of RMSSD and SDNN across the different body position conditions (supine and seated) and devices (H10 vs. OH1) using intraclass correlation coefficients (ICC), mean absolute error (MAE), root mean square error (RMSE), and Bland–Altman analysis.

Variables	Conditions (1 vs. 2)	Mean ± SD (1 vs. 2)	ICC (95% CI)	MAE	RMSE	Mean Diff.	Lower LoA	Upper LoA
**RMSSD** **5 min** **(ms)**	*H10sup.* vs. *OH1sup.*	37 ± 21 vs. 41 ± 20	0.955 (0.911–0.978)	4.32	6.09	−3.21	−13.51	7.09
	*H10seat.* vs. *OH1seat.*	36 ± 25 vs. 44 ± 23	0.834 (0.699–0.912)	8.33	13.92	−8.05	−30.69	14.59
	*H10sup.* vs. *H10seat.*	37 ± 21 vs. 36 ± 25	0.608 (0.338–0.786)	13.99	19.85	1.22	−38.25	40.69
	*OH1sup.* vs. *OH1seat.*	41 ± 20 vs. 44 ± 23	0.560 (0.270–0.756)	14.38	20.23	−3.61	−43.28	36.05
**RMSSD** **2 min** **(ms)**	*H10sup.* vs. *OH1sup.*	36 ± 18 vs. 39 ± 21	0.869 (0.757–0.931)	5.64	9.98	−2.91	−21.93	16.12
	*H10seat.* vs. *OH1seat.*	34 ± 22 vs. 41 ± 20	0.868 (0.756–0.931)	6.89	10.72	−6.14	−23.65	11.37
	*H10sup.* vs. *H10seat.*	36 ± 18 vs. 34 ± 22	0.482 (0.169–0.707)	13.12	19.90	1.35	−38.19	40.88
	*OH1sup.* vs. *OH1seat.*	39 ± 21 vs. 41 ± 20	0.468 (0.145–0.701)	13.46	20.95	−1.89	−43.47	39.69
**SDNN** **5 min** **(ms)**	*H10sup.* vs. *OH1sup.*	51 ± 22 vs. 54 ± 22	0.980 (0.959–0.990)	2.72	4.32	−2.11	−9.62	5.40
	*H10seat.* vs. *OH1seat.*	54 ± 31 vs. 60 ± 29	0.921 (0.848–0.960)	6.43	11.96	−6.02	−26.60	14.57
	*H10sup.* vs. *H10seat.*	51 ± 22 vs. 54 ± 31	0.728 (0.545–0.845)	14.00	19.54	−2.02	−40.75	36.72
	*OH1sup.* vs. *OH1seat.*	54 ± 22 vs. 60 ± 29	0.674 (0.454–0.817)	15.44	20.90	−5.92	−45.86	34.02
**SDNN** **2 min** **(ms)**	*H10sup.* vs. *OH1sup.*	45 ± 21 vs. 48 ± 22	0.929 (0.861–0.964)	4.89	7.96	−2.63	−17.60	12.34
	*H10seat.* vs. *OH1seat.*	51 ± 31 vs. 56 ± 29	0.916 (0.833–0.957)	6.77	12.28	−5.23	−27.36	16.90
	*H10sup.* vs. *H10seat.*	45 ± 21 vs. 51 ± 31	0.621 (0.392–0.778)	13.79	22.56	−5.32	−48.99	38.35
	*OH1sup.* vs. *OH1seat.*	48 ± 22 vs. 56 ± 29	0.507 (0.223–0.712)	17.04	25.92	−7.92	−57.09	41.25

**Table 3:** Comparison of RMSSD and SDNN between age groups (≤40 vs. >40 years) across the different body position conditions (supine and seated) and devices (H10 vs. OH1) using intraclass correlation coefficients (ICC), mean absolute error (MAE), root mean square error (RMSE), and Bland–Altman analysis.

Variables	Conditions (1 vs. 2)	Mean ± SD (1 vs. 2)	ICC (95% CI)	MAE	RMSE	Mean Diff.	Lower LoA	Upper LoA
**RMSSD** **≤40 years old** **(ms)**	*H10sup.* vs. *OH1sup.*	42 ± 21 vs. 44 ± 23	0.981 (0.944–0.994)	3.17	4.22	−1.94	−9.89	6.01
	*H10seat.* vs. *OH1seat.*	35 ± 16 vs. 38 ± 15	0.951 (0.870–0.982)	3.31	4.47	−3.31	−9.91	3.29
	*H10sup.* vs. *H10seat.*	42 ± 21 vs. 35 ± 16	0.584 (0.207–0.810)	14.11	17.60	7.28	−22.39	36.95
	*OH1sup.* vs. *OH1seat.*	44 ± 23 vs. 38 ± 15	0.585 (0.228–0.804)	12.93	17.35	5.91	−25.16	36.99
**RMSSD** **>40 years old** **(ms)**	*H10sup.* vs. *OH1sup.*	31 ± 18 vs. 36 ± 17	0.912 (0.787–0.965)	5.27	7.27	−4.47	−16.06	7.12
	*H10seat.* vs. *OH1seat.*	36 ± 31 vs. 49 ± 28	0.812 (0.594–0.919)	12.45	18.36	−12.08	−40.01	15.85
	*H10sup.* vs. *H10seat.*	31 ± 18 vs. 36 ± 31	0.623 (0.335–0.805)	13.89	21.52	−5.11	−47.34	37.13
	*OH1sup.* vs. *OH1seat.*	36 ± 17 vs. 49 ± 28	0.581 (0.286–0.775)	15.57	22.33	−12.72	−49.80	24.37
**SDNN** **≤40 years old** **(ms)**	*H10sup.* vs. *OH1sup.*	55 ± 17 vs. 56 ± 19	0.977 (0.941–0.991)	1.89	3.68	−1.08	−8.55	6.39
	*H10seat.* vs. *OH1seat.*	53 ± 17 vs. 55 ± 18	0.984 (0.957–0.994)	2.46	2.96	−2.46	−6.22	1.30
	*H10sup.* vs. *H10seat.*	55 ± 17 vs. 53 ± 17	0.598 (0.129–0.849)	12.43	14.57	1.97	−28.46	32.39
	*OH1sup.* vs. *OH1seat.*	56 ± 19 vs. 55 ± 18	0.624 (0.167–0.860)	12.26	15.08	0.58	−31.17	32.33
**SDNN** **>40 years old** **(ms)**	*H10sup.* vs. *OH1sup.*	48 ± 25 vs. 51 ± 25	0.980 (0.949–0.992)	3.40	4.79	−3.01	−10.53	4.52
	*H10seat.* vs. *OH1seat.*	53 ± 40 vs. 62 ± 37	0.912 (0.787–0.965)	9.70	15.92	−9.07	−35.50	17.36
	*H10sup.* vs. *H10seat.*	48 ± 25 vs. 53 ± 40	0.754 (0.542–0.875)	15.31	22.84	−5.50	−50.30	39.30
	*OH1sup.* vs. *OH1seat.*	51 ± 25 vs. 62 ± 37	0.693 (0.423–0.850)	18.05	24.69	−11.57	−55.63	32.49

**Table 4:** Comparison of RMSSD and SDNN between males and females across the different body position conditions (supine and seated) and devices (H10 vs. OH1) using intraclass correlation coefficients (ICC), mean absolute error (MAE), root mean square error (RMSE), and Bland–Altman analysis.

Variables	Conditions (1 vs. 2)	Mean ± SD (1 vs. 2)	ICC (95% CI)	MAE	RMSE	Mean Diff.	Lower LoA	Upper LoA
**RMSSD** **Female** **(ms)**	*H10sup.* vs. *OH1sup.*	29 ± 13 vs. 33 ± 11	0.851 (0.678–0.934)	4.66	6.41	−3.76	−14.24	6.72
	*H10seat.* vs. *OH1seat.*	29 ± 12 vs. 37 ± 10	0.521 (0.196–0.743)	8.30	11.93	−8.27	−25.62	9.07
	*H10sup.* vs. *H10seat.*	29 ± 13 vs. 29 ± 12	0.450 (−0.002–0.750)	9.17	12.49	0.59	−24.57	25.75
	*OH1sup.* vs. *OH1seat.*	33 ± 11 vs. 37 ± 10	0.251 (−0.198–0.613)	9.49	12.90	−3.92	−28.70	20.86
**RMSSD** **Male** **(ms)**	*H10sup.* vs. *OH1sup.*	48 ± 25 vs. 51 ± 26	0.974 (0.921–0.991)	3.86	5.60	−2.46	−12.73	7.82
	*H10seat.* vs. *OH1seat.*	46 ± 34 vs. 54 ± 32	0.870 (0.646–0.956)	8.36	16.28	−7.74	−36.96	21.49
	*H10sup.* vs. *H10seat.*	48 ± 25 vs. 46 ± 34	0.555 (0.076–0.826)	20.66	26.89	2.09	−52.61	56.79
	*OH1sup.* vs. *OH1seat.*	51 ± 26 vs. 54 ± 32	0.524 (0.010–0.819)	21.15	27.31	−3.19	−58.53	52.15
**SDNN** **Female** **(ms)**	*H10sup.* vs. *OH1sup.*	41 ± 11 vs. 43 ± 11	0.939 (0.851–0.976)	2.49	3.86	−2.13	−8.62	4.35
	*H10seat.* vs. *OH1seat.*	42 ± 12 vs. 50 ± 13	0.513 (0.145–0.757)	7.19	13.09	−7.07	−29.28	15.14
	*H10sup.* vs. *H10seat.*	41 ± 11 vs. 42 ± 12	0.317 (−0.157–0.672)	10.22	13.28	−1.33	−27.98	25.31
	*OH1sup.* vs. *OH1seat.*	43 ± 11 vs. 50 ± 13	−0.031 (−0.424–0.373)	14.61	18.28	−6.28	−40.90	28.35
**SDNN** **Male** **(ms)**	*H10sup. vs OH1sup.*	66 ± 25 vs. 68 ± 26	0.980 (0.937–0.993)	3.03	4.89	−2.09	−11.11	6.93
	*H10seat.* vs. *OH1seat.*	69 ± 42 vs. 73 ± 39	0.967 (0.902–0.989)	5.37	10.19	−4.55	−23.15	14.04
	*H10sup.* vs. *H10seat.*	66 ± 25 vs. 69 ± 42	0.702 (0.419–0.860)	19.26	25.82	−2.96	−55.29	49.36
	*OH1sup.* vs. *OH1seat.*	68 ± 26 vs. 73 ± 39	0.720 (0.409–0.881)	16.58	24.07	−5.43	−53.26	42.40

## Data Availability

The original data and Matlab codes used in the study are openly available in H10_OH1_analysis at https://github.com/GeoffreyMillour/H10_OH1_analysis.git (accessed on 1 September 2025).

## Abbreviations

The following abbreviations are used in this manuscript:

ANS	Autonomic nervous system
ECG	Electrocardiography
HR	Heart rate
HRV	Heart rate variability
LoA	Limits of agreement
PAT	Pulse arrival time
PPG	Photoplethysmography
PPI	Peak-to-peak interval
PTT	Pulse transit time
PRV	Pulse rate variability
RMSSD	Root mean square of successive differences
SDNN	Standard deviation of normal-to-normal intervals
